# Containing COVID-19: Implementation of Early and Moderately Stringent Social Distancing Measures Can Prevent The Need for Large-Scale Lockdowns

**DOI:** 10.5334/aogh.2969

**Published:** 2020-07-29

**Authors:** Wee Chian Koh, Mohammad Fathi Alikhan, David Koh, Justin Wong

**Affiliations:** 1 Centre for Strategic and Policy Studies, Brunei Darussalam, BN; 2 Disease Control Division, Ministry of Health, Brunei Darussalam, BN; 3 PAPRSB Institute of Health Sciences, Universiti Brunei Darussalam, BN; 4 Saw Swee Hock School of Public Health, National University of Singapore, BN

## Abstract

Guidance from many health authorities recommend that social distancing measures should be implemented in an epidemic when community transmission has already occurred. The clinical and epidemiological characteristics of COVID-19 suggest this is too late. Based on international comparisons of the timing and scale of the implementation of social distancing measures, we find that countries that imposed early stringent measures recorded far fewer cases than those that did not. Yet, such measures need not be extreme. We highlight the examples of Hong Kong and Brunei to demonstrate the early use of moderate social distancing measures as a practical containment strategy. We propose that such measures be a key part of responding to potential future waves of the epidemic.

## Introduction

On March 12, 2020, UK Prime Minister Boris Johnson, in a speech outlining the government’s decision to move the country from containment to the delay phase of the COVID-19 response, announced that “At all stages, we have been guided by the science, and we will *do the right thing at the right time* [emphasis added].” [1] In a strategy that has now been severely criticized, the United Kingdom waited and did not close schools, ban sporting events, or other large gatherings.

Despite the current consensus that the United Kingdom dithered, the evidence for the type, scale, and timing of social distancing measures in managing COVID-19 is unclear. Social distancing refers to measures that aim to decrease or interrupt disease transmission by minimizing physical contact between potentially infected and healthy individuals. Many health authorities recommend the implementation of such measures at a fairly late stage in a local epidemic, that is when community transmission has already occurred [2]. On the other hand, models estimate that had enhanced social distancing measures been introduced one week, two weeks, or three weeks earlier in China, the number of COVID-19 cases could have been reduced by 66%, 86%, and 95%, respectively [3]. A report from the United States suggests that 90% of cumulative deaths could have been avoided had social distancing measures been implemented two weeks earlier [4].

In order to review how measures were introduced across various countries, we compare the timing and scale of the implementation of social distancing measures. We then highlight the examples of Brunei and Hong Kong to demonstrate the real-world feasibility of early use of social distancing measures in controlling COVID-19. Implementing such measures early on need not be unnecessarily disruptive. We propose that the adoption of moderate social distancing measures at a very early stage of the epidemic is practical as a containment strategy, preventing the need for “lockdowns” at a later stage.

## International Comparisons

**Figure 1:**
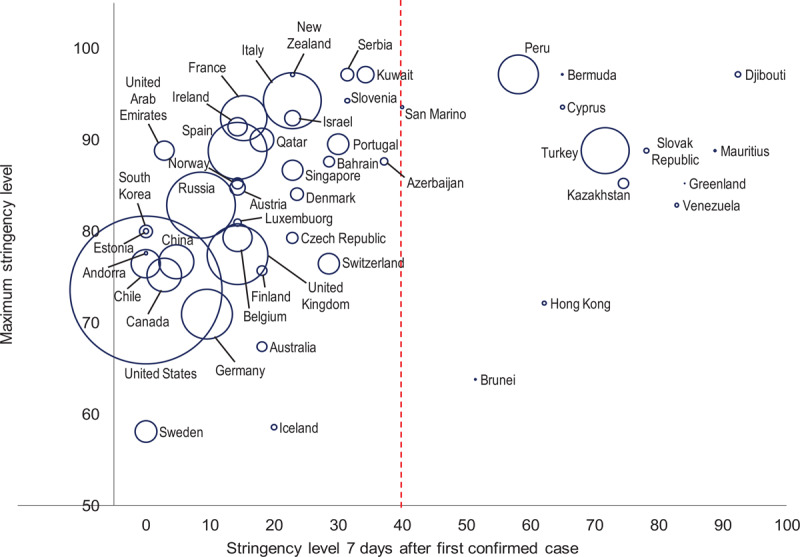
Total COVID-19 cases and government responses. Source: Oxford COVID-19 Government Response Tracker, Worldometer COVID-19 Tracker, Our World in Data, official government sources. *Note*: The stringency level is a composite index of nine policy measures: school closing; workplace closing; cancel public events; restrictions on gatherings; close public transport; stay-at-home requirements; restrictions on internal movement; international travel restrictions; and public info campaigns. The index ranges from 0 to 100. A higher index indicates a higher stringency. The size of a bubble is proportional to a country’s total number of cases. Countries below a tests-per-million threshold of 20,000 are excluded to reduce under-detection bias, with the exception of China. A similar picture is observed with the total number of deaths. Data as of May 20, 2020.

We assess the response of countries by their stringency, measured as a composite index of government responses to COVID-19: school closing; workplace closing; cancel public events; restrictions on gatherings; close public transport; stay-at-home requirements; restrictions on internal movement; international travel restrictions; and public info campaigns [5]. The importance of early stringent measures is illustrated in Figure 1. The horizontal axis shows a country’s stringency level seven days after recording the first case. The vertical axis is the maximum stringency level reached (as of May 20). The size of a bubble is proportional to a country’s total number of cases. Only countries that have significant testing efforts (more than 20,000 tests per million people) are included to minimize bias caused by under-detection.

Countries to the right of the red dashed line—those with early stringent interventions—recorded a much lower number of cases (fewer than 2,000 as of May 20, except for Turkey, Peru, and Kazakhstan). Conversely, countries to the left—those with less stringent measures early on—have had severe outbreaks, such as China, Italy, Spain, the United Kingdom, and the United States. These countries were eventually forced into more drastic social distancing interventions, including lockdowns of entire communities. The specific implementation of such lockdowns varies across different countries, but usually involve mass quarantine, closing non-essential services, and in some cases, the application of cordon sanitaire.

There are some notable exceptions, however. Mass testing and tracing in Estonia and Iceland helped achieve encouraging outcomes but may not be feasible in countries that lack the institutional capacity for nationwide contact tracing. New Zealand and Slovenia implemented full lockdowns before the surge in cases became unmanageable, but this approach is likely to be costly and unacceptable to many. In fact, the feasibility of lockdown approaches may be influenced by the principal economic activities of the respective countries [6]. For example, high-income countries predominantly dominated by high-tech industries can more easily switch to remote working differently from low-income countries where economic activities are trade and agriculture.

On the other hand, Peru and Turkey are among the hardest hit countries despite early intervention, highlighting the importance of complementary measures. Peru announced a full lockdown nine days after detecting its first case, but a limited social safety net and historically weaker public institutions meant that it could not secure community compliance [7]. Turkey’s response to the pandemic should be seen within the wider context of extreme levels of political and societal polarization. While the government restricted mass gatherings fairly early, public messaging has been inconsistent, and other more severe restrictions have been implemented abruptly, causing mass panic. Turkey was also relatively slow in restricting travel from Iran (the second epicenter outside China) and did not quarantine returning pilgrims from Mecca until significant public outcry [8].

## COVID-19 Containment In Early Movers

Hong Kong and Brunei appear to be outliers in Figure 1, as the only two countries in the lower right quadrant. They did not resort to extreme measures, demonstrating that containment is feasible at a moderate level of stringency, and suggesting the possibility of a threshold of effective public health intervention [9].

Hong Kong, an international travel hub and among the densely populated cities in the world, implemented border control and social distancing early, including school closures and working from home. Its community also spontaneously adopted personal protective behaviors such as mask wearing [10]. Aggressive contact tracing, and quarantine of close contacts of confirmed cases was also undertaken. Despite sharing a border with mainland China and detecting its first case on January 22, Hong Kong has done relatively well in limiting the spread of COVID-19 to 1,055 cases (May 20) without the need for a lockdown, particularly when compared to New Zealand (1,154 cases and three-fifths of Hong Kong’s population) [11].

The early success of Brunei, a small country in Southeast Asia, owes much to its containment strategy through case detection and ring fencing of cases and their contacts [12]. However, these measures alone are insufficient to control disease spread [13]. Over a 10-day period from the onset of Brunei’s first case on March 9, a series of measures were implemented: school closures, the prohibition of mass gatherings, mosque closures, and international travel restrictions. However, public services and businesses remain open, and no movement restrictions within the country were imposed. These interventions—stringent, but not drastic—appear to have paid off: as of May 20, Brunei has recorded only 141 cases. By contrast, Iceland—with three-quarters the population of Brunei—has almost 13 times more cases. Moreover, the maximum stringency level for Brunei at 63.8 is not much higher than Sweden (generally considered to have the least stringent response in Europe) at 58.1. The difference is that Brunei, unlike Sweden, implemented these measures very early on in its outbreak.

In both Brunei and Hong Kong, the approach to social distancing measures has been non-binary. High-risk settings and activities such as travel and mass gatherings, particularly in enclosed spaces, were restricted, however lower risk activities such as outdoor recreational activities that can be conducted with relevant safety measures in place have continued. Implementing moderate social distancing measures based on understanding the continuum of transmission risk can be crucial for securing greater community compliance and a more sustainable response.

## Earlier Is Better

Why did so many countries leave it until it was too late? Many health authorities have developed pandemic preparedness plans based on experience from SARS and influenza. These recommend that social distancing measures should be implemented when: (i) extensive transmission of the virus is ongoing; (ii) a significant number of cases lack an epidemiological link; (iii) quarantine of contacts is no longer sufficient to prevent further spread [13].

We suggest that in the case of COVID-19, the above criteria are too late for three main reasons. First, most patients have mild or asymptomatic disease [14]. Second, combining the estimated serial interval of around four days with an incubation period of 5–6 days suggests the potential for pre-symptomatic or asymptomatic transmission [15,16]. Third, greater viral shedding during the early phase suggests very high transmissibility, evident in the attack rates on the cruise ship Diamond Princess [17].

Given that many countries employ testing criteria based on the presence of symptoms, by the time community transmission is first detected, it may already be widespread, with multiple silent chains of transmission well-established [18]. Model-based estimates show that, with a basic reproduction number (R_0_) of 2.5, about 70% of close contacts have to be successfully traced to control early spread, which is unlikely given the limited case detection strategies employed in many countries [19].

As such, even with the best efforts at testing, case identification, and quarantine, the potential for widespread community transmission is clear. Once established, suppression necessitates the implementation of severely disruptive social distancing measures [20]. China had to issue the largest quarantine in history to control the outbreak. As the pandemic unfolds, it becomes apparent that the short-term cost of early stringent measures will be far lower than the long-term cost of reactive interventions.

## Limitations

Our analysis has several limitations. Principally, our model looks solely at aggregated de jure measures for social distancing interventions without taking into account other potential determinants of COVID-19 transmission. Other studies show an association between increasing temperature and slower growth of COVID-19 cases [21], potentially accounting for the early successes of Brunei and Hong Kong in comparison with countries such as Iceland. Travel plays a significant role in driving transmission dynamics – importation events in a country with no cases can lead to an exponential increase in the case of numbers within a short time period [22]. Our model aggregates international travel restrictions with other social distancing measures but does not assess the individual impact of international travel, which could potentially play an outsize role in relation to non-pharmaceutical interventions given many countries vulnerabilities to importation and exportation events. Finally, the explosive growth of COVID-19 models other emerging epidemics with a high proportion of cases driven by superspreading events [23]. The impact of these events and the influence of general social distancing measures in mitigating outbreaks, particularly in healthcare and other residential, institutional settings, should also be assessed.

## Conclusion

The success of Hong Kong and Brunei in controlling the initial waves of the COVID-19 epidemic without imposing draconian measures illustrates how swift and decisive social distancing measures can support an aggressive case finding and contact tracing strategy. In this respect, “the right time” is earlier than conventionally accepted. Extreme measures, as observed in many countries, could possibly be averted had moderately stringent social distancing interventions been imposed early.

The fight is far from over. Modeling studies suggest the possibility of multiple waves before the end of the year [24,25]. As countries in lockdown begin to reach a level of suppression and start developing exit strategies, we are of the view that the application of early and moderate social distancing measures is a key part of responding to potential future waves of the epidemic. These measures help keep the outbreak in check, enabling the core strategy of testing, tracing, and isolation to succeed while at the same time, allowing optimal care for infected patients.
